# Data for the prevalence of nurses׳ burnout in Iran (a meta-analysis dataset)

**DOI:** 10.1016/j.dib.2018.09.022

**Published:** 2018-09-11

**Authors:** Alireza Khammar, Sahar Dalvand, Amir Hossein Hashemian, Mohsen Poursadeghiyan, Soudabeh Yarmohammadi, Jalal Babakhani, Hamed Yarmohammadi

**Affiliations:** a Department of Occupational Health Engineering, School of Health, Zabol University of Medical Sciences, Zabol, Iran; b Health Promotion Research Center, Iran University of Medical Sciences, Tehran, Iran; c Research Center for Environmental Determinants of Health (RCEDH), Kermanshah University of Medical Sciences, Kermanshah, Iran; d Health in Emergency and Disaster Research Center, University of Social Welfare and Rehabilitation Sciences, Tehran, Iran; e Department of Health Education and Health Promotion, School of Public Health, Shahid Beheshti University of Medical Sciences, Tehran, Iran; f Department of Nursing, Kermanshah University of Medical Sciences, Kermanshah, Iran

**Keywords:** Burnout, Nurse, Iran, Systematic review, Meta-analysis

## Abstract

The present dataset was carried out using meta-analysis method towards investigation of the prevalence of nurses׳ burnout in Iran. To this end, the keywords were searched in the Iranian databases such as Medlib, SID, Iranmedex, Magiran or even some international databases such as Cochrane, Science-Direct, Scopus, PubMed, and Google Scholar. The data were analysed using the STATA Software Version 12. In ten articles with a sample size of 1758 subjects, an average age of 30.73 (54%) and the confidence interval of 43–64, the prevalence of burnout was reported. The obtained data indicated that Fars and Zanjan Provinces had the highest and lowest rates of burnout (72% and 26%, respectively). According to the acquired data, the total prevalence of burnout among men and women measured 46% and 65%, respectively. Given the high prevalence of burnout among the Iranian nurses in this dataset and the importance of nursing in public health which requires highly motivated and committed nurses with high job satisfaction, it is recommended that the intensity of burnout be reduced through supervising the nurses׳ professional performance, supporting, paying attention to their problems, following up and providing the necessary strategies to improve their environmental, economic, and personal conditions.

**Specifications table**

**Table:** 

Subject area	Nursing and Health Professions
More specific subject area	Occupational Health
Type of Data	Tables and Figures
How data was acquired	The data of was related to a meta-analysis research. Moreover, the English and Persian articles were extracted from the Iranian database, such as Medlib, SID, Iranmedex, Magiran, and from other valid international databases such as Cochrane, Science-Direct, Scopus, PubMed, and Google Scholar. Furthermore, the STATA Software Version 12.0 was utilized to analyse the raw data.
Data Format	Raw Data and Analyzed data
Experimental Factors	The point estimation and a confidence interval of 95% were calculated for the prevalence of burnout in each assessment through considering the variables of gender and geographical areas.
Experimental Features	A form was used for data extraction with the following variables: number of samples, type of study, age, geographical area, city or province, population, the total prevalence of burnout, burnout in men and women, sample size, name of the authors and the year of publication.
Location of Data Source	Kermanshah, Iran
Data Accessibility	Data were included in this article
Related research article	J. Adriaenssens, V. De Gucht, S. Maes, Determinants and prevalence of burnout in emergency nurses: A systematic review of 25 years of research, Int. J. Nurs. Stud. 52, 2015, 649–61 [1].

**Value of the data**

• The obtained data of this dataset can be employed in further studies to investigate the prevalence of burnout among nurses in Iran.
• The data of the present dataset was related to prevalence of nurses׳ burnout in throughout Iran. Therefore, the data obtained are more valuable than studies that are done individually.
• This dataset can be useful for provide various strategies to reduce the prevalence of nurses׳ burnout in Iran.
• The method of data collection in this study is meta-analysis. Therefore, this methodology can be useful for future similar studies.

## 1 Data

**Fig. 1:**
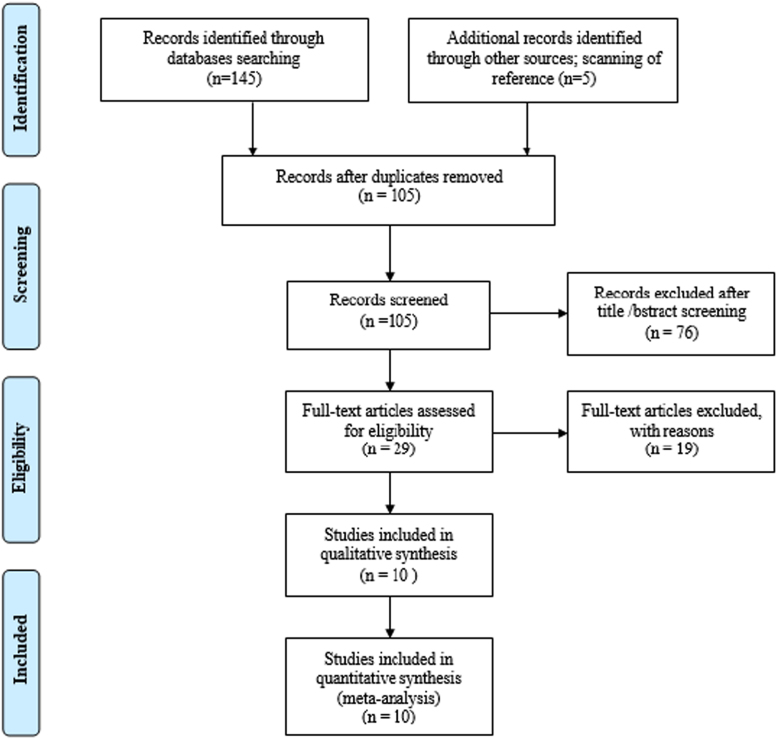
Flow chart of the study and selection of articles based on the PRISMA steps.

Based on the inclusion criteria, out of 145 studies using the Maslach questionnaire to assess the prevalence of burnout, 10 cross-sectional studies were entered into the meta-analysis process [3], [4], [5], [6], [7], [8], [9], [10], [11], [12] (Fig. 1). The sample size included 1758 subjects with an average of 175 subjects per study. In Table 1, the specifications of the selected studies are presented.

**Table 1:** The Specifications of the articles in a systematic review and meta-analysis of prevalence of burnout among the Iranian nurses.

**Author**	**Year**	**Sample size**	**Male**	**Female**	**Province**	**Prevalence of burnout**	**95% CI**	
							**Lower limit**	**Upper limit**
Saheb-Zamani et al. [2]	2008	93	41	52	Tehran	64	55	74
Rouhi et al. [3]	2008	272	95	177	Golestan	54	48	60
Ghaedi et al. [4]	2011	120	60	60	Guilan	35	27	44
Jamali-Mogahdam and Soleimani [5]	2010	114	–	–	Fars	68	59	76
Mohammadi et al. [6]	2002	400	54	346	Ardabil	66	62	71
Ziaei et al. [7]	2013	189	74	115	Kermanshah	47	40	54
Payami Bousary [8]	2002	151	30	121	Zanjan	26	19	33
Shafaghat et al. [9]	2016	245	25	220	Fars	75	70	81
Hosseiniarzfuni et al. [10]	2015	120	70	50	Mazandaran	54	45	63
Khajeddin et al. [11]	2003	54	38	16	Tehran	44	31	58

**Fig. 2:**
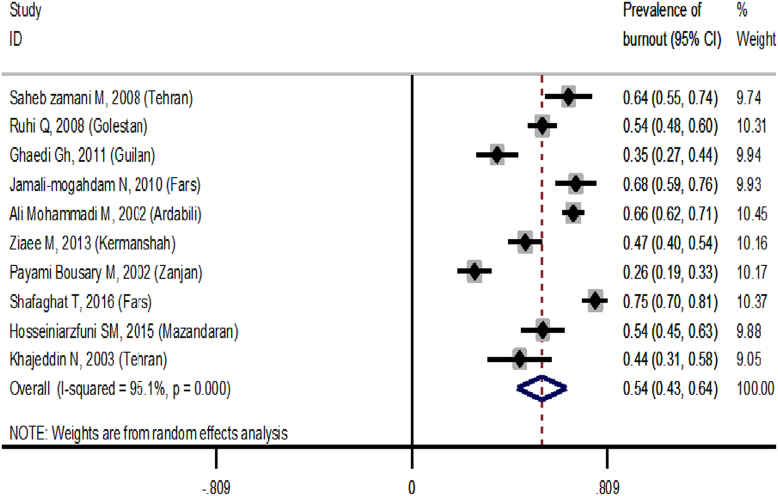
The prevalence of burnout based on the database.

**Fig. 3:**
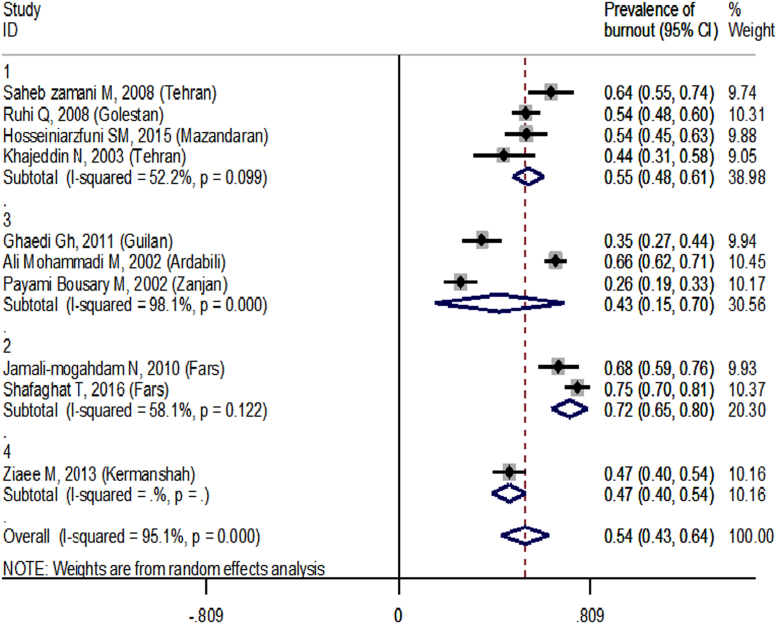
The prevalence of burnout based on the geographical regions. *Region 1***:** Alborz, Tehran, Qazvin, Mazandaran, Semnan, Golestan, and Qom. *Region 2***:** Esfahan, Fars, Bushehr, Hormozgan, Kohgiluyeh and Boyer-Ahmad, and Chaharmahal and Bakhtiari. *Region 3*: West Azerbaijan, East Azerbaijan, Ardabil, Zanjan, Gilan, and Kurdistan. *Region 4***:** Kermanshah, Ilam, Lorestan, Hadaman, Markazi, and Khuzestan. *Region 5***:** Razavi Khorasan, North Khorasan, South Khorasan, Kerman, Yazd, and Sistan and Baluchestan.

The prevalence of burnout based on the database and geographical regions are showed in Figs. 2 and 3, respectively. The findings demonstrated that the overall prevalence of burnout measured 54% (95% CI: 43–64). Based on subgroup analysis, the highest and lowest rates of burnout were reported in the area 2 (72%) and 3 (43%), respectively (Fig. 3).

**Fig. 4:**
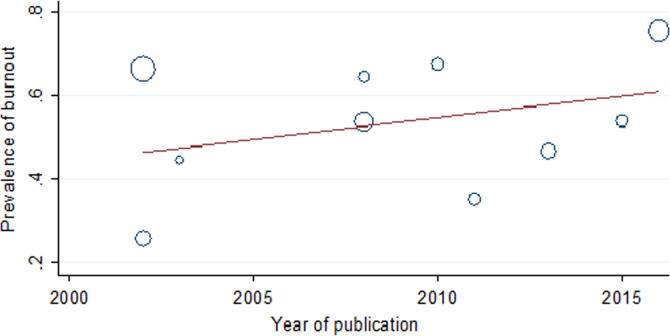
The meta-regression plot of the prevalence of burnout based on the year of publication.

**Fig. 5:**
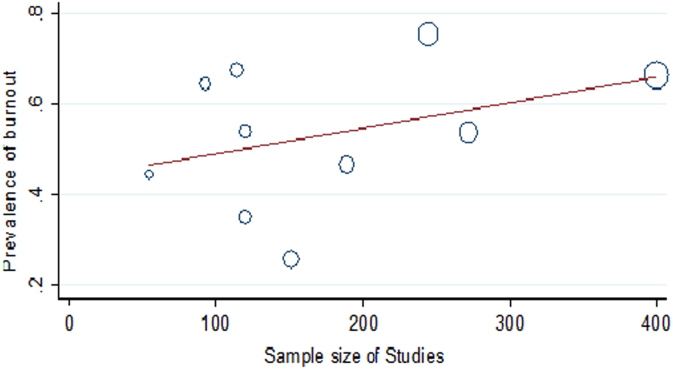
The meta-regression plot of the prevalence of burnout based on sample size.

Based on the results of the meta-regression test, although the frequency of burnout increased in line with the years of conducting the studies, sample size and mean of age of subjects, this growing trend were not statistically significant ( Figs. 4 and 5). The obtained data of funnel plot indicated there was no publication bias in the present dataset.

**Fig. 6:**
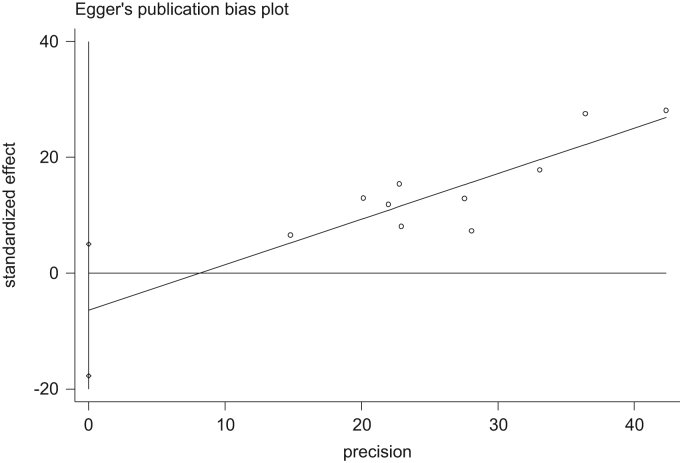
The funnel plot of the analyzed studies.

**Fig. 7:**
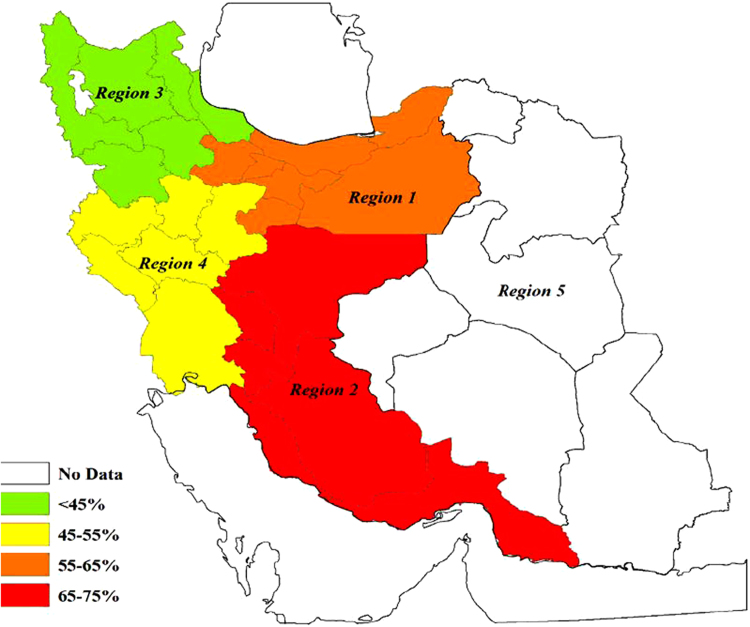
The overall prevalence of burnout in different geographical regions. This map was created using the ARCGIS software by ESRI (http://www.esri.com).

The univariate meta-regression data for the prevalence of burnout, the years of conducting the studies, sample size and mean of age of nurses in Iran are showed in Table 2. Additionally, the funnel plot of the investigated studies and the overall prevalence of burnout in different geographical regions are presented in Figs. 6 and 7, respectively.

**Table 2:** The univariate meta-regression results for the prevalence of burnout, the years of conducting the studies, sample size and mean of age of nurses in Iran.

**Variable**	**Coefficient**	**SE**	***t***	**Confidence interval**		***p***
				**Lower limit**	**Upper limit**	
Year	0.0104	0.0101	1.03	−0.0129	0.0337	0.333
Sample size	0.0010	0.001	1.13	−0.0010	0.0017	0.292
Mean age	0.0142	0.063	0.23	−0.786	0.815	0.859

## 2 Experimental design, materials and methods

### 2.1 Search strategy

In this data article, the prevalence of burnout in Iranian nurses was reviewed based on the published studies without time limitations until December 2016. To this end, the keywords were searched in the Iranian databases such as Medlib, SID, Iranmedex, Magiran or even some international databases such as Cochrane, Science-Direct, Scopus, PubMed, and Google Scholar. The sources of related articles were also reviewed for access to other articles.

### 2.2 Inclusion and exclusion criteria

All articles addressing the prevalence of burnout in nursing staff using the Maslach Questionnaire were collected [2], [3], [4], [5], [6], [7], [8], [9], [10], [11]. The studies were selected based on inclusion and exclusion criteria. The exclusion criteria included: non-relevant studies (reviews, editorials, non-research letters), studies with non-random sampling method, case reports, interventional studies, insufficient data, duplicate publications, not using the Maslach Questionnaire to assess the prevalence of burnout, the prevalence of burnout in other healthcare groups, and lack of access to the full text of studies.

### 2.3 Data extraction

To reduce the bias, the search of articles was independently done by two researchers, and in the event of disagreement, the study was judged by another expert in meta-analysis (DS). Then, the required information such as the title of the article, the first author, year of publication, prevalence of burnout, place of study, total sample size, sample size by gender, mean age of participants, geographical regions and province of studies were collected from the selected articles, and the prevalence of burnout was recorded in a form, too. The articles’ screening and selection process was conducted according to the PRISMA Guidelines [12].

### 2.4 Data analysis

The point estimation and a confidence interval of 95% were calculated for the prevalence of burnout in each study using the Der Simonian and Laird׳s random effects model. Moreover, the Cochran *Q* test and I^2^ index were used to investigate the heterogeneity between studies

To evaluate the small effects of the study and potential population bias, a funnel plot was used based on the Egger Regression Test. In addition, to study the relationship between the prevalence of burnout and each of the years of conducting the studies and the sample size, a meta- regression analysis was used. Further, the subgroups analysis was applied to estimate the prevalence of burnout in each geographical region. As for data analysis, the STATA Software Version 12.0 was employed (Stata Corp, College Station, and TX).

## References

[1] Adriaenssens J., De Gucht V., Maes S. (2015). Determinants and prevalence of burnout in emergency nurses: a systematic review of 25 years of research. Int. J. Nurs. Stud..

[2] Saheb-Zamani M., Safavi M., Farahani H. (2009). Burnout of nurses employed at Tehran psychiatric hospitals and its relation with social supports. Med. Sci. J..

[3] Rouhi Q., Einollah M., Mahmoodi G. (2008). The management approach of nurse administrators and occupational burnout among nurse׳s staff of Golestan University hospitals. J. Jahrom Univ. Med. Sci..

[4] Ghaedi G., Mohammadipour R.N., Ghasemi M.H. (2015). Comparison of fixed and rotating shifts on burnout among nurses working in public and private hospitals in Rasht. Daneshvar Med..

[5] Jamali-Mogahdam N., Soleimani S. (2017). Burnout and its relationship with social support of nursing in hospitals of Shiraz University of Medical Sciences, 2010. Sadra Med. Sci. J..

[6] Mohammadi M.A., Dadkhah B., Mozaffari A., Dostkami H. (2007). Survey of burnout among nurses working in hospitals in Ardabil. J. Health Care.

[7] Ziaei M., Yarmohammadi H., Karamimatin B., Yarmohammadi S., Nazari Z., Gharagozlou F. (2014). Prevalence and risk factors of occupational burnout among nurses of a hospital in Kermanshah in 2013. J. Ergon..

[8] Payami Bousary M. (2002). Burnout syndrome in nurses, working at educational hospitals. Iran. J. Nurs..

[9] Shafaghat T., Rahimi-Zarchi M.K., Kavosi Z. (2016). Studying the status of job burnout and its relationship with demographic characteristics of nurses in Shiraz Nemazee Hospital. Hosp. Pract. Res..

[10] Hosseiniarzfuni S.M., Gorji A.M., Ranjabar M., Giorji R.H., Rostamnejad M. (2015). Job burnout among technicians and nurses of Northern Iran. Gaziantep Med. J..

[11] Khajeddin N., Hakim Shoushtari M., Hajebi A. (2016). The impact of perception of locus of control on burnout syndrome among nurses in a psychiatric hospital. Iran. J. Psychiatry Clin. Psychol..

[12] Moher D., Liberati A., Tetzlaff J., Altman D.G. (2010). Preferred reporting items for systematic reviews and meta-analyses: the PRISMA statement. Int. J. Surg..

